# A descriptive study of youth risk behavior in urban and rural secondary school students in El Salvador

**DOI:** 10.1186/1472-698X-6-3

**Published:** 2006-04-11

**Authors:** Andrew E Springer, BJ Selwyn, Steven H Kelder

**Affiliations:** 1 Center for Health for Health Promotion and Prevention Research, University of Texas School of Public Health, The University of Texas Health Science Center at Houston, Houston, Texas, USA; 2 Division of Management, Policy and Community Health,, University of Texas School of Public Health, The University of Texas Health Science Center at Houston, Houston, Texas, USA

## Abstract

**Background:**

Adolescence is an important stage of life for establishing healthy behaviors, attitudes, and lifestyles that contribute to current and future health. Health risk behavior is one indicator of health of young people that may serve both as a measure of health over time as well as a target for health policies and programs. This study examined the prevalence and distribution of youth health risk behaviors from five risk behavior domains–aggression, victimization, depression and suicidal ideation, substance use, and sexual behaviors–among public secondary school students in central El Salvador.

**Methods:**

We employed a multi-stage sampling design in which school districts, schools, and classrooms were randomly selected. Data were collected using a self-administered questionnaire based on the United States Center for Disease Control and Prevention's Youth Risk Behavior Survey. Sixteen schools and 982 students aged 12–20 years participated in the study.

**Results:**

Health risk behaviors with highest prevalence rates included: engagement in physical fight (32.1%); threatened/injured with a weapon (19.9%); feelings of sadness/hopelessness (32.2%); current cigarette use (13.6%); and no condom use at last sexual intercourse (69.1%). Urban and male students reported statistically significant higher prevalence of most youth risk behaviors; female students reported statistically significant higher prevalence of feelings of sadness/hopelessness (35.6%), suicidal ideation (17.9%) and, among the sexually experienced, forced sexual intercourse (20.6%).

**Conclusion:**

A high percentage of Salvadoran adolescents in this sample engaged in health risk behaviors, warranting enhanced adolescent health promotion strategies. Future health promotion efforts should target: the young age of sexual intercourse as well as low condom use among students, the higher prevalence of risk behaviors among urban students, and the important gender differences in risk behaviors, including the higher prevalence of reported feelings of sadness, suicidal ideation and forced sexual intercourse among females and higher sexual intercourse and substance use among males. Relevance of findings within the Salvadoran and the cross-national context and implications for health promotion efforts are discussed.

## Background

Adolescence is an important stage of life for establishing healthy behaviors, attitudes, and lifestyles that contribute to current and future health. In 2002, the United Nations Special Session on Children entitled 'A World Fit for Children' underscored the importance of this by recommending that all countries establish national policies and programs, including goals and indicators, to promote adolescent health [1]. Youth risk behavior, a general term used to describe adverse health behaviors adopted in childhood or adolescence, is one indicator of the health of young people that serves as a basis for measuring adolescent health over time as well as a target for health policies and programs. The importance of this measure is based on its association with several mortality and morbidity outcomes, including intentional injury stemming from aggression and suicidal ideation, chronic disease resulting from substance use and misuse, sexually transmitted disease, and such undesired social outcomes as unintended teenage pregnancy [2]

Despite the important connection between health behavior and overall health, many countries still lack basic prevalence estimates of youth risk behavior. To our knowledge, no comprehensive study on youth risk behavior among Salvadoran adolescents has been published to date. Our research reports the prevalence of selected youth risk behaviors (aggressive behavior, victimization, substance use, depression and suicidal ideation, and sexual behaviors) from a representative cross-sectional sample of Salvadoran secondary students attending rural and urban public schools in the central region of El Salvador. In addition to exploring differences of risk behavior prevalence within subgroups of this population for the purpose of focusing intervention efforts, this study provides baseline data that can be used to measure youth risk behavior in this region of El Salvador over time.

## Methods

### Sampling design

El Salvador is divided administratively into 14 departments (geographical administrative divisions similar to states or provinces). The present study was carried out in a department located in the central region of the country that had among the highest population concentrations after San Salvador and one of the lowest proportions of households in poverty (33%; 41% of households in poverty for total country) at the time of the study [3]. The sampling frame for the study consisted of 19 of the 20 school districts in this department. These 19 districts participated in the "model school" program (*escuelas modelo*), an initiative by the Ministry of Education aimed to provide innovative teaching techniques and school/classroom organization. Based on the potential for differences in risk behavior among students attending the model and regular schools, we incorporated both school types into the sampling design.

The public school system in El Salvador includes primary school, secondary school, and *bachillerato *(similar to upper level high school). Secondary school comprises grades 7 through 9, with students in the target age ranges of 12 through 15 years. For this study, a multi-stage sampling design was employed in which districts, secondary schools, and classrooms were randomly selected. The first sampling stage consisted of randomly selecting 8 out of the 19 districts. Each district had one model school that was designated as urban or rural by the Ministry of Education; approximately half of the districts were represented by a rural model school and half by an urban model school. The districts were stratified by urban or rural classification of the model school, with equal numbers of rural and urban schools selected for the study. The second sampling stage consisted of randomly selecting one regular school from the total number of regular schools within the districts that matched the rural/urban classification of the model school. Within each of the 16 urban/rural and model/regular schools selected for the study, the final stage of sampling consisted of randomly selecting one eighth- and one ninth-grade classroom. Schools had one or two eighth and ninth grade classes with an average class size of 31 students.

### Procedures

Using a self-administered questionnaire, data were collected June through August of 1999. The questionnaire was administered in classrooms according to standardized data collection procedures by a team comprising a technician from the National Training Center of the Ministry of Education of El Salvador, a Salvadoran university student, and the principal investigator (AS).

An oral and written description of the study detailing the subject matter and placing specific emphasis on the study's voluntary and confidential nature was provided to all potential participants. In addition, passive parental informed consent for participation in the study was obtained for all participants. At the time of the research, passive parental informed consent was an accepted protocol of the United States Centers for Disease Control and Prevention (CDC) in Atlanta, Georgia, for conducting research with adolescents in their *Youth Risk Behavior Survey *(YRBS), a national monitoring tool for assessing health risk behavior trends [4]. This consent procedure consists of sending a letter home with the student that describes the study and asks parents or guardians to submit a signed form only if they do not wish their child to participate in the study. This protocol, along with the study objectives, methods, and English language version of the questionnaire, was reviewed and approved by the Committee for the Protection of Human Subjects of the University of Texas Health Science Center at Houston; the Spanish translation of the study objectives, methods and questionnaire were reviewed and approved by a review team composed of Salvadoran parents and representatives of the Ministry of Education of El Salvador.

### Questionnaire development and measures

The self-administered questionnaire consisted of closed-ended items with dichotomous and ordinal level response choices to assess health risk behaviors and school context. All items were translated from English to Spanish and back-translated and centered with the assistance of two Salvadoran university students and a native English speaker. The risk behavior study items were reviewed for content and appropriateness within the Salvadoran context by national-level Ministry of Education officials. In order to determine the level of comprehension of the items, the questionnaire was pilot-tested with a separate sample of 35 urban eighth-grade students attending a school in the study area.

#### Youth risk behavior measures

All items measuring youth risk behaviors were selected from the instrument used for the U.S. Youth Risk Behavior Survey from the CDC [5], a comprehensive adolescent health survey that has been administered biennially in the United States since 1991 [4]. The risk behavior items comprised five behavioral domains: aggressive behavior, assessed by four items related to physical fighting and weapon carrying; victimization, measured by four items about having been threatened or injured with a weapon in the past 12 months, having felt too unsafe to go to school in past 30 days, having been hit or physically hurt by partner in past 12 months ('*pareja*', i.e. boyfriend or girlfriend), and having ever been forced to have sexual intercourse against his/her will; depressive symptoms and suicidal ideation in the past 12 months, measured by three items about feeling sad or hopeless for two or more weeks in a row, seriously considering attempting suicide, and having previously attempted suicide; substance use, measured by four questions relating to episodic heavy alcohol drinking (having drunk ≥ 5 alcoholic drinks in a row) in past 30 days, lifetime and current cigarette use (having smoked 1 or more cigarettes in past 30 days), and lifetime marijuana use (having ever smoked marijuana); and sexual and reproductive behavior, assessed by four items about ever having had sexual intercourse, having had sexual intercourse at or before age 13, use of condoms at last sexual intercourse, and ever having been or gotten someone pregnant. Although the CDC YRBS also includes questions related to dietary behaviors and physical activity, this survey focused on the five behavioral domains described above based on the principal causes of mortality in El Salvador at the time of the survey (see discussion below) as well as the need to limit the length of the questionnaire. All risk behavior variables were dichotomized (yes/no) based on classifications of risk behaviors from the CDC Youth Risk Behavior Survey [6].

#### Demographics and other classifying characteristics

We also studied the association of youth risk behaviors in relation to gender, age, subjective economic status, and geographic location of school (independent variables). Student age ranged from 12 to 20 years, and students were divided into three groups based on developmental considerations: 12–14 years, an important developmental stage that differs from older adolescence [7]; 15–16 years; and 17–20 years. Subjective economic status was based on a single item measured on a 5-point response scale with 1 = living very well off to 5 = poor. This measure was adapted from the Gore, Aseltine, and Colton [8] study of social structure, life stress, and depressive symptoms among United States high school students. Based on conceptual similarities of the response categories, we dichotomized this variable into those living very well off and living comfortably ("high" subjective economic status), and those who are just getting by, almost poor, and poor ("low" subjective economic status). Geographic location of school (rural vs. urban) was determined according to school classifications from the Ministry of Education of El Salvador. In addition to the independent variables, the study assessed potential confounding with the variable of school type (model vs. regular).

### Analysis

Data were edited to eliminate or resolve errors, checked for logical inconsistencies, and entered into the database. All analyses were carried out with the statistical software package Stata 9.0 (College Station, Texas) and were based on the pooled sample of eighth- and ninth-grade students. Following criteria used in the U.S. Youth Risk Behavior analysis [6], differences between prevalence estimates were considered statistically significant if p < .05. We evaluated the bivariate relations of the independent variables mentioned above with the dependent youth risk behavior variables by calculating prevalence odds ratios and 95% confidence intervals. Stratified analyses were conducted to assess subgroup comparisons of urban and rural students by gender groups with the risk behavior variables.

Multivariable logistic regression was performed to assess the effect of each independent variable while controlling for gender, age, subjective economic status, and geographic classification of school. As preliminary analyses found no statistically significant differences in risk behavior between students attending regular and model schools (p > .10 for all risk behaviors) [data not shown], students were pooled for the multivariate analyses. Adjusted estimates for the final models are based on the same set of covariates in order to allow comparability among the five risk behavior domains, and listwise deletion was used to handle missing cases.

## Results

All 16 schools (2 from each school district) agreed to participate in the study. With 17 students opting not to participate in the study and 8 questionnaires deemed ineligible due to invalid or inconsistent responses (e.g., contradictory responses, such as stating 'never smoked' and then responding 'smoked 20 or more times'), the total sample size of 982 students represents a response rate of 97.5%. Approximately half of the students were from rural areas (50.2%), and half attended model schools (49.3%) [data not shown]. For both rural and urban schools, just over half of the total sample was male (~ 52%). The ages of the study participants ranged from 12–20 years, with a mean age (SD) of 15.25 (1.42) years. While students in the younger age groups were equally distributed by gender, males represented a higher proportion of the oldest age group (63.2%). A higher percentage of urban students (64.2%) compared to rural students (53.9%) reported a subjective economic status of "living well off/living comfortably".

### Aggressive behavior

Among all study participants, physical fighting was the highest reported aggression-related variable, with 32.1% reporting to have been in a physical fight within the previous 12 months (Table 1). Male and urban students were more likely to report each of the four violence-related behaviors; however, no statistically significant differences were found between urban and rural students for carrying a weapon in the previous 30 days. Age was not significantly associated with any of the aggression measures.

**Table 1:** Prevalence of aggressive behavior among eighth and ninth grade secondary students attending public schools in a central region of El Salvador by socio-demographic characteristics, 1999

Factor	Sample Size	Phyisical fight in past 12 months		Physical fight at school in past 12 months		Carried a weapon in past 30 days		Carried a weapon at school in past 30 days	
					
		% (CI)^c^	Adj. OR (95% CI)^d^	% (CI)^c^	Adj. OR (95% CI)^d^	% (CI)^c^	Adj. OR (95% CI)^d^	% (CI)^c^	Adj. OR (95% CI)^d^
*Gender*									
Male	516	41.9 (± 3.1)	**2.78 (2.04, 3.72)**	22.5 (± 2.6)	**1.95 (1.36, 2.78)**	20.5 (± 2.5)	**8.29 (4.56, 15.07)**	8.4 (± 1.7)	**4.16 (1.98, 8.76)**
Female^a^	463	21.4 (± 2.6)	1.00	13.0 (± 2.1)	1.00	3.0 (± 1.1)	1.00	1.9 (± 0.9)	1.00
*Age Group *(in years)									
12 to 14^a^	292	33.6 (± 3.0)	1.00	19.7 (± 2.6)	1.00	11.0 (± 2.0)	1.00	6.1 (± 1.5)	1.00
15 to 16	467	30.6 (± 3.0)	0.94 (0.68, 1.31)	17.7 (± 2.5)	0.95 (0.64, 1.40)	12.0 (± 2.1)	1.15 (0.71, 1.86)	4.9 (± 1.4)	0.85 (0.44, 1.63)
17 to 20	173	32.9 (± 3.0)	1.04 (0.68, 1.59)	16.8 (± 2.4)	0.93 (0.55, 1.55)	14.0 (± 2.2)	1.16 (0.64, 2.12)	3.5 (± 1.2)	0.56 (0.21, 1.47)
*Subjective Economic Status*^b^									
Low	390	33.1 (± 3.0)	1.08 (0.80, 1.45)	17.5 (± 2.4)	1.00 (0.70, 1.43)	14.2 (± 2.2)	1.29 (0.85, 1.96)	6.2 (± 1.5)	1.41 (0.77, 2.58)
High^a^	564	32.4 (± 3.0)	1.00	18.8 (± 2.5)	1.00	11.2 (± 2.0)	1.00	5.0 (± 1.4)	1.00
*School Geography*									
Urban	489	40.5 (± 3.1)	**2.15 (1.59, 2.89)**	24.1 (± 2.7)	**2.31 (1.61, 3.32)**	13.5 (± 2.1)	1.41 (0.92, 2.16)	7.4 (± 1.6)	**2.65 (1.36, 5.15)**
Rural^a^	490	23.9 (± 2.7)	1.00	11.9 (± 2.0)	1.00	10.9 (± 2.0)	1.00	3.3 (± 1.1)	1.00
Total	979	32.1 (± 2.9)	N/A	18.0 (± 2.4)	N/A	12.2 (± 2.1)	N/A	5.3 (± 1.4)	N/A

Bold text indicates significant at < .05 level.

Abbreviations: ref, referent; Subj. E.S., Subjective Economic Status; Prev., Prevalence; Adj. OR, Adjusted Odds Ratio; CI, 95% Confidence Interval; N/A, not applicable.

^a^Referent group;^b^Low = self-classification as "poor", "nearly poor" or "just getting by"; High = self-classification as "living very well off" or "living comfortably."^c^Prevalence is based on bivariate association and is unadjusted.^d^OR adjusted for all factors in table

Stratified analyses of rural and urban students by gender [data not shown in tables] revealed that urban males were more likely to report physical fighting in the previous 12 months in general and at school (Prevalence Odds Ratio [POR] = 2.43, 95% Confidence Interval [CI]: 1.67, 3.54 and POR = 2.74, 95% CI: 1.73, 4.37, respectively) and carrying a weapon in previous 30 days at school (POR = 2.16, 95% CI: 1.05, 4.48) than their rural male counterparts. As with urban males, urban females were more likely than rural females to report physical fighting in general and at school (POR = 2.02, 95% CI: 1.25, 3.28, and POR = 1.89, 95% CI: 1.04, 3.44, respectively). No gender-specific differences between urban and rural students were observed for carrying a weapon in the previous 30 days.

### Victimization

For the four victimization measures, the highest prevalence was reported for having been threatened or injured with a weapon in the previous 12 months (19.9% of students) (Table 2). Additionally, 7.5–10.1% of students reported ever having been forced to have sexual intercourse, having been hit or physically hurt by partner, and having felt too unsafe to go to school. Males were more likely to report having been threatened or injured with a weapon and for feeling too unsafe to go to school; no statistically significant gender differences were observed for having been hit or physically hurt by partner. Female students were almost four times as likely to report ever having been forced to have sexual intercourse against will. While urban students tended to report higher prevalence of all victimization behaviors compared to rural students, having been threatened or injured with a weapon was the only victimization behavior in which urban students were significantly different than rural students. Students with lower subjective economic status were 1.80 times as likely to report feeling unsafe to go to school in the past 30 days (95% CI: 1.16, 2.79).

**Table 2:** Prevalence of victimization among eighth and ninth grade secondary students attending public schools in a central region of El Salvador by socio-demographic characteristics, 1999

Factor	Sample Size	Threatened or injured with weapon in past 12 months		Felt too unsafe to go to school in past 30 days		Hit or physically hurt by partner in past 12 months		Ever forced to have sexual intercourse against will^e^	
					
		% (CI)^c^	Adj. OR (95% CI)^d^	% (CI)^c^	Adj. OR (95% CI)^d^	% (CI)^c^	Adj. OR (95% CI)^d^	% (CI)^c^	Adj. OR (95% CI)^d^
*Gender*									
Male	515	27.0 (± 2.8)	**2.95 (2.06, 4.23)**	12.3 (± 2.1)	**1.64 (1.05, 2.56)**	8.8 (± 1.8)	1.40 (0.83, 2.35)	6.1 (± 3.0)	1.00
Female	464	11.9 (± 2.0)	1.00	7.6 (± 1.7)	1.00	6.0 (± 1.5)	1.00	20.6 (± 5.1)	**3.85 (1.30, 11.42)**
*Age Group*(in years)									
12 to 14^a^	293	17.4 (± 2.4)	1.00	10.0 (± 1.9)	1.00	6.8 (± 1.7)	1.00	3.3 (± 1.2)	1.00
15 to 16	465	20.4 (± 2.6)	1.26 (0.85, 1.86)	10.3 (± 2.3)	1.07 (0.65, 1.77)	7.2 (± 1.7)	1.11 (0.61, 2.01)	7.3 (± 1.7)	0.99 (0.28, 3.53)
17 to 20	172	23.8 (± 2.7)	1.47 (0.90, 2.39)	10.5 (± 2.0)	1.02 (0.53, 1.94)	8.8 (± 1.9)	1.33 (0.64, 2.75)	10.5 (± 2.0)	1.28 (0.32, 5.13)
*Subjective Economic Status *^b^									
Low	388	23.2 (± 2.7)	1.30 (0.92, 1.83)	13.7 (± 2.2)	**1.80 (1.16, 2.79)**	9.1 (± 1.9)	1.44 (0.87, 2.41)	6.9 (± 1.7)	1.15 (0.41, 3.26)
High	563	17.9 (± 2.4)	1.00	7.8 (± 1.7)	1.00	6.7 (± 1.6)	1.00	6.0 (± 1.6)	1.00
*School Geography*									
Urban	489	23.4 (± 2.7)	**1.70 (1.20, 2.39)**	11.5 (± 2.0)	1.38 (0.89, 2.15)	8.6 (± 1.8)	1.59 (0.95, 2.68)	7.5 (± 1.7)	1.44 (0.51, 4.04)
Rural^a^	490	16.4 (± 2.3)	1.00	8.7 (± 1.8)	1.00	6.4 (± 1.6)	1.00	5.6 (± 1.5)	1.00
Total	979	19.9 (± 2.5)	N/A	10.1 (± 1.9)	N/A	7.5 (± 1.6)	N/A	8.1 (± 3.4)	N/A

Bold text indicates significant at < .05 level.

Abbreviations: ref, referent; Prev., Prevalence; Adj. OR, Adjusted Odds Ratio; CI, 95% Confidence Interval; N/A, not applicable.

^a^Referent group; ^b^Low = self-classification as "poor", "nearly poor" or "just getting by"; High = "living very well off" or "living comfortably"; ^c^Prevalence is based on bivariate association and is unadjusted; ^d^OR adjusted for all factors in table; ^e^Among those reporting sexual intercourse (n = 246).

In exploring geographic differences by gender [data not shown in tables], we found that urban males were 1.86 times as likely to report having been threatened or injured with a weapon in the past year (95% CI: 1.23, 2.82) and 1.85 times as likely to report feeling too unsafe to go to school (95% CI: 1.06, 2.79) as rural males. Urban females were 2.55 times as likely to report having been hit or physically hurt by partner in the past 12 months as rural females (95% CI: 1.03, 6.50).

### Depressive symptoms and suicidal ideation

Approximately one-third of students reported having felt so sad or hopeless every day for two or more weeks in a row that they stopped doing some usual activities (Table 3). A notable percentage of students also reported having seriously considered suicide (12.7%) and having attempted suicide (7.6%). Females were significantly more likely to report feelings of sadness, suicidal ideation, and having attempted suicide. Students of lower subjective economic status and from urban areas were more likely to report feelings of sadness and suicidal ideation. Students aged 17–20 years were significantly more likely to report feelings of sadness/hopelessness than students 12–14 years. While age was not significantly associated with the suicide measures, prevalence of seriously considering suicide and suicide attempt increased with the oldest age group.

**Table 3:** Prevalence of depressive symptoms and suicidal ideation among eighth & ninth grade secondary students attending public schools in a central region of El Salvador by socio-demographic characteristics, 1999

Factor	Sample Size	Felt sad or hopeless every day for 2 weeks in a row^c^		Seriously considered attempting suicide^c^		Attempted suicide^c^	
				
		% (CI)^d^	Adj. OR (95% CI)^e^	% (CI)^d^	Adj. OR (95% CI)^e^	% (CI)^d^	Adj. OR (95% CI)^e^
*Gender*							
Male^a^	512	29.1 (± 2.9)	1.00	8.0 (± 1.7)	1.00	5.1 (± 1.4)	1.00
Female	463	35.6 (± 3.0)	**1.47 (1.11, 1.96)**	17.9 (± 2.4)	**2.83 (1.87, 4.30)**	10.4 (± 1.9)	**2.51 (1.49, 4.23)**
*Age Group*(in years)							
12 to 14^a^	294	28.6 (± 2.9)	1.00	12.8 (± 2.1)	1.00	7.2 (± 1.7)	1.00
15 to 16	462	30.7 (± 3.0)	1.16 (0.83, 1.61)	12.4 (± 2.1)	0.95 (0.60, 1.49)	7.8 (± 1.7)	1.11 (0.62, 1.99)
17 to 20	174	41.4 (± 3.2)	**1.98 (1.31, 2.99)**	15.0 (± 2.3)	1.41 (0.80, 2.50)	8.6 (± 1.8)	1.42 (0.69, 2.93)
*Subjective Economic Status *^b^							
Low	388	37.4 (± 3.1)	**1.42 (1.06, 1.90)**	16.4 (± 2.4)	**1.88 (1.26, 2.82)**	9.1 (± 1.8)	1.44 (0.87, 2.38)
High^a^	561	29.6 (± 2.9)	1.00	10.5 (± 2.0)	1.00	6.6 (± 1.6)	1.00
*School Geography*							
Urban	486	36.2 (± 3.0)	**1.57 (1.18, 2.10)**	15.3 (± 2.3)	**1.76 (1.17, 2.65)**	8.1 (± 1.7)	1.22 (0.74, 2.01)
Rural^a^	489	28.2 (± 2.8)	1.00	10.1 (± 1.9)	1.00	7.2 (± 1.6)	1.00
Total	976	32.2	N/A	12.7	N/A	7.6	N/A

Bold text indicates significant at < .05 level.

Abbreviations: ref, referent; Prev., Prevalence; Adj. OR, Adjusted Odds Ratio; CI, 95% Confidence Interval; N/A, not applicable.

^a^Referent group; ^b^Low = self-classification as "poor", "nearly poor" or "just getting by"; High = "living very well off" or "living comfortably"; ^c^During 12 months preceding survey; ^d^Prevalence is based on bivariate association and is unadjusted; ^e^OR adjusted for all factors in table.

When we looked at geographic differences stratified by gender [data not shown in tables], we saw no statistically significant differences between urban and rural males for the depression and suicide measures. While urban females were more likely than rural females to report feelings of sadness or hopelessness (POR = 1.54, 95% CI: 1.03, 2.29), no statistically significant differences were observed for suicidal ideation and suicide attempt.

### Substance use

Approximately half of the male students and 19.4% of female students reported having tried cigarettes in their lifetimes (Table 4). Current cigarette use was notably high for males, with roughly one out of five reporting to have smoked at least one cigarette in the previous month. Students reported much lower lifetime marijuana use (4.9% for total sample). Prevalence of episodic heavy drinking fell between cigarette use and marijuana use, with 9.8% of the sample reporting this behavior. Males were significantly more likely to report cigarette and marijuana use; no statistically significant gender differences were observed for episodic heavy alcohol drinking. For all four substance use measures, prevalence increased with age. Students aged 17–20 years were roughly two times as likely as the youngest age group to report episodic heavy drinking and current cigarette use; no statistically significant age differences were observed with lifetime marijuana use. Urban students were significantly more likely to report lifetime and current cigarette use.

**Table 4:** Prevalence of selected substance abuse behaviors among eighth and ninth grade secondary students attending public schools in a central region of El Salvador by socio-demographic characteristics, 1999

Factor	Sample Size	Episodic heavy drinking^c^		Lifetime cigarette use^f^		Current cigarette use^g^		Lifetime marijuana use^h^	
					
		% (CI)^d^	Adj. OR (95% CI)^e^	% (CI)^d^	Adj. OR (95% CI)^e^	% (CI)^d^	Adj. OR (95% CI)^e^	% (CI)^d^	Adj. OR (95% CI)^e^
*Gender*									
Male	514	11.5 (± 2.0)	1.45 (0.93, 2.28)	45.1 (± 3.1)	**3.51 (2.59, 4.76)**	19.1 (± 2.5)	**2.82 (1.84, 4.34)**	6.7 (± 1.6)	**2.13 (1.12, 4.07)**
Female^a^	462	8.0 (± 1.7)	1.00	19.4 (± 2.5)	1.00	7.4 (± 1.6)	1.00	3.0 (± 1.1)	1.00
*Age Group*(in years)									
12 to 14^a^	292	6.2 (± 1.5)	1.00	29.7 (± 2.9)	1.00	9.9 (± 1.9)	1.00	4.5 (± 1.3)	1.00
15 to 16	467	11.1 (± 2.0)	**2.01 (1.15, 3.54)**	33.0 (± 3.0)	1.27 (0.91, 1.79)	13.6 (± 2.2)	1.60 (0.99, 2.59)	5.0 (± 1.4)	1.19 (0.59, 2.42)
17 to 20	171	12.3 (± 2.1)	**2.31 (1.17, 4.54)**	39.5 (± 3.1)	**1.60 (1.04, 2.46)**	19.9 (± 2.6)	**2.46 (1.39, 4.34)**	5.8 (± 1.5)	1.37 (0.57, 3.27)
*Subjective Economic Status *^b^									
Low	390	10.3 (± 1.9)	0.96 (0.61, 1.51)	34.4 (± 3.0)	0.99 (0.74, 1.34)	14.7 (± 2.3)	1.12 (0.75, 1.67)	5.4 (± 1.4)	1.12 (0.61, 2.06)
High^a^	560	9.8 (± 1.9)	1.00	33.0 (± 3.0)	1.00	13.2 (± 2.2)	1.00	4.8 (± 1.4)	1.00
*School Geography*									
Urban	487	11.5 (± 2.0)	1.51 (0.96, 2.37)	38.1 (± 3.1)	**1.79 (1.33, 2.41)**	17.5 (± 2.4)	**2.60 (1.71, 3.94)**	6.2 (± 1.5)	1.80 (0.97, 3.37)
Rural^a^	489	8.2 (± 1.7)	1.00	28.6 (± 2.8)	1.00	9.7 (± 1.9)	1.00	3.7 (± 1.2)	1.00
Total	976	9.8 (± 1.9)	N/A	33.3 (± 3.0)	N/A	13.6 (± 2.2)	N/A	4.9 (± 1.4)	N/A

Bold text indicates significant at < .05 level.

Abbreviations: ref, referent; Prev., Prevalence; Adj. OR, Adjusted Odds Ratio; CI, 95% Confidence Interval; N/A, not applicable.

^a^Referent group; ^b^Low = self-classification as "poor", "nearly poor" or "just getting by"; High = "living very well off" or "living comfortably"; ^c^Drank 5 drinks of alcohol in a row on 1 of the 30 days preceding the survey; ^d^Prevalence is based on bivariate association and is unadjusted; ^e^OR adjusted for all factors in table; ^f^Ever tried cigarette smoking;^g^smoked cigarettes on 1 of the 30 days preceding survey; ^h^Ever used marijuana.

No statistically significant differences were found for episodic drinking between urban males and females and their rural counterparts [data not shown in tables]. On the other hand, urban males were more likely to report both lifetime and current cigarette use than rural males (POR = 1.42, 95% CI: 1.02, 2.11 and POR = 1.78, 95% CI: 1.11, 2.36, respectively), and urban females were more likely to report lifetime and current cigarette use than rural females (POR = 1.89, 95% CI: 1.14, 3.13 and POR = 2.98, 95% CI: 1.29, 7.06, respectively). No significant differences in lifetime marijuana use were observed between urban male and females and their rural counterparts.

### Sexual behavior

Males were significantly more likely to report ever having had sexual intercourse (just under half) as were females (8%), and males were more likely to report having had their first intercourse at or before 13 years of age (Table 5). While sexual intercourse increased with age, prevalence of sexual intercourse at or before 13 years of age was highest among the youngest age group and decreased with age. No differences in sexual behaviors were observed by subjective economic status or geographic location for the total sample. In exploring geographic differences stratified by gender [data not shown], urban males were more likely than rural males to report having had sexual intercourse at or before age 13 (POR = 1.99, 95% CI: 1.14, 3.49); no differences were reported between urban and rural females. With regard to protective behaviors, 30% of sexually experienced males and 34% of sexually experienced females reported using a condom during their last sexual intercourse. Lastly, 7% of sexually experienced males reported having gotten someone pregnant; no females in the study reported having been pregnant.

**Table 5:** Prevalence of selected sexual and reproductive behaviors among eighth and ninth grade secondary students attending public schools in a central region of El Salvador by socio-demographic characteristics, 1999

		Ever had sexual intercourse			First intercourse age 13		No condom use at last sexual intercourse		Had been pregnant or gotten someone pregnant	
						
Factor	n	% (CI)^c^	Adj. OR (95% CI)^d^	n	% (CI)^c^	Adj. OR (95% CI)^d^	% (CI)^c^	Adj. OR (95% CI)^d^	% (CI)^c^	Adj. OR (95% CI)^d^
*Gender*										
Male	510	45.3 (± 3.1)	**10.92 (7.25, 16.44)**	230	50.7 (± 6.0)	**3.45 (1.40, 8.51)**	69.6 (± 5.5)	1.14 (0.52, 2.53)	7.2 (± 3.1)	N/A^e^
*Female*^a^	463	7.8 (± 1.7)	1.00	35	28.6 (± 5.4)	1.00	65.7 (± 5.7)	1.00	0	
*Age Group *(in years)										
12 to 14^a^	292	18.5 (± 2.5)	1.00	54	79.6 (± 5.0)	1.00	75.5 (± 5.3)	1.00	3.7 (± 2.4)	1.00
15 to 16	464	26.1 (± 2.8)	**1.85 (1.24, 2.76)**	121	43.0 (± 6.1)	**0.20 (0.09, 0.43)**	67.2 (± 5.8)	0.66 (0.32, 1.39)	2.6 (± 2.0)	0.68 (0.11, 4.26)^f^
17 to 20	171	46.2 (± 3.2)	**4.62 (2.83, 7.54)**	77	28.6 (± 5.6)	**0.11 (0.05, 0.25)**	68.8 (± 5.8)	0.68 (0.30, 1.54)	14.1 (± 4.3)	3.62 (0.72, 18.15)^f^
Subjective Economic Status^b^										
Low	387	27.4 (± 2.8)	0.73 (0.51, 1.03)	105	42.9 (± 6.0)	0.90 (0.51, 1.60)	68.9 (± 5.6)	0.98 (0.55, 1.73)	7.8 (± 3.3)	0.99 (0.33, 2.92)
High^a^	561	28.2 (± 2.9)	1.00	158	50.6 (± 6.0)	1.00	69.5 (± 5.6)	1.00	5.3 (± 2.8)	1.00
*School Geography*										
Urban	483	28.6 (± 2.8)	1.35 (0.96, 1.90)	138	54.3 (± 6.0)	1.59 (0.91, 2.77)	67.0 (± 5.7)	0.82 (0.47, 1.43)	3.7 (± 2.3)	0.55 (0.17, 1.71)
Rural^a^	490	26.3 (± 2.8)	1.00	127	40.2 (± 5.9)	1.00	71.0 (± 5.5)	1.00	8.9 (± 3.5)	1.00
Total	973	27.4 (± 2.8)	N/A	265	47.5 (± 6.0)	N/A	69.1 (± 5.6)	N/A	6.6 (± 3.0)	N/A

Bold text indicates significant at < .05 level.

Abbreviations: ref, referent; Prev., Prevalence; Adj. OR, Adjusted Odds Ratio; CI, 95% Confidence Interval; N/A, not applicable.

^a^Referent group; ^b^Low = self-classification as "poor", "nearly poor" or "just getting by"; High = "living very well off" or "living comfortably"; ^c^Prevalence is based on bivariate association and is unadjusted; ^d^OR adjusted for all factors in table; ^e^Not calculated as prevalence for females = 0; ^f^includes males only

## Discussion

Several of the leading causes of mortality in El Salvador can be related to various health risk behaviors, including behaviors related to unintentional and intentional injuries and tobacco and other drug use. In 2004, gun-related violence, motor vehicle accidents, cardiovascular disease, mental health and alcohol use-related complications were among the top ten causes of mortality in El Salvador [9]. In adolescents between the ages of 15 and 19, leading causes of mortality include intentional injuries (homicide and suicide), unintentional injuries, cardiovascular disease, and complications during birth [10].

In contributing to an understanding of health risk behavior in El Salvador, the present study examined the prevalence and distribution of selected youth health risk behaviors among a sample of public secondary school students in a central department of El Salvador. We found that 20–30% of Salvadoran secondary school students reported engagement in at least one health risk behavior from each of the five domains examined and that the prevalence of specific health risk behaviors varied considerably depending upon gender, geographic location of school, and, to a lesser extent, age and subjective economic status. In addition to the immediate health risks posed by engagement in specific risk behaviors [2], some adolescent health risk behaviors have been found to track into adulthood [11,12]. The large percentage of students engaging in risk behavior found in this study along with immediate and future implications of adolescent health risk behavior underscore the continued need to create the conditions for health promoting behaviors in adolescents in El Salvador.

### Magnitude of problem

Youth risk behavior prevalence estimates for U.S. Hispanics of similar age from the 1999 U.S. YRBS [2,13] serve as a comparison for interpreting the magnitude of Salvadoran youth risk behavior prevalence. We found that Salvadoran youth were comparable with U.S. Hispanic youth in their reporting of aggressive and victimization behaviors, feelings of sadness, suicidal ideation and suicide attempts, and pregnancy history for males. Substance use, on the other hand, was lower for Salvadoran youth, with selected risk behaviors of binge drinking and lifetime marijuana use reported by 10% and 5%, respectively, of Salvadoran youth (mean age 15.25 years) and 25% and 40% for U.S. Hispanic youth of comparable age. Although U.S. Hispanic youth aged 15 years reported higher prevalence for ever having had sexual intercourse than Salvadoran youth (44% compared with 27%, respectively), sexually experienced Salvadoran youth reported lower condom use at last sexual intercourse than U.S. Hispanic youth (31% and 69%, respectively) and higher prevalence of sexual intercourse at or before 13 years of age (48% and 42%, respectively).

While comparisons of adolescent health risk behavior prevalence between Latin American countries are challenging due to the use of different measures, distinct age distributions, and the small but growing number of studies, selected studies from Latin America with similar age groups may provide insights into the magnitude of prevalence of specific risk behaviors examined in this study. The combined gender prevalence of 33% of lifetime cigarette use for Salvadoran adolescents in this study compares with a combined gender prevalence of between 20–47% in the PACARDO study of five Central American countries, Panama and the Dominican Republic [14]. In a study of 2,059 Brazilian adolescents, eighth grade Brazilian male students reported a substantially lower prevalence of current cigarette smoking (8%) [15] compared to the 19% reported for Salvadoran males in this study. The 4.9% combined prevalence of marijuana use found for girls and boys in this study approximates the average 4% marijuana use found for the seven Latin American countries in the PACARDO study [14].

Our finding of a higher prevalence of self-reported sexual intercourse at a young age for males follows similar gender patterns for early age at sexual initiation observed for adolescents in Colombia [16], Brazil [15] and the Dominican Republic [17]. As more studies on adolescent risk behaviors in Latin America become available, a systematic review comparing prevalence of key risk behaviors may provide important insights into the cultural, contextual and policy-related factors that promote or inhibit adolescent health risk behavior in Latin America.

### Gender differences

In line with findings from the U.S. Youth Risk Behavior Survey for 1999 [2], we found important gender differences in the distribution of youth risk behaviors reported by Salvadoran students. Male students were significantly more likely than female students to report all aggression-related behaviors as well as two of the four victimization behaviors–*having been threatened or hurt with a weapon *and *feeling too unsafe to go to school*. This gender difference for engagement in violence-related behavior is reflected in the larger Salvadoran population, where 70–80% of homicides occur among males, with more than half occurring among males aged 15–30 years [18]. The 20% of male adolescents who reported carrying a weapon in the past 30 days along with the over 40% who reported participating in a physical fight in the past year may be causes for concern given that El Salvador has been reported to have the second highest homicide rate among young people in the Americas [19].

We found a high percentage of sexually experienced females (20%) reported that they had been forced to have sexual intercourse, and female students were almost four times as likely to report having been forced to have sexual intercourse as males. Heise and colleagues [20] reported that between one-third and two-thirds of known sexual assault victims are age 15 or younger based on justice system statistics and rape crisis centers in eight countries, which included the U.S. and four Latin American countries. In neighboring Nicaragua, one study found that 26% of women between the ages of 25 and 44 reported that they had experienced sexual abuse before the age of 19, and 15% reported having been victims of attempted or completed rape–a rate twice that of men [21]. While nongovernmental organizations in El Salvador such as CEMUJER and governmental institutions such as the Instituto Salvadoreño para el Desarrollo de la Mujer (Salvadoran Institute for the Development of Women)–established in 1996 to develop and ensure the implementation of a national policy on women's development and rights– are signs of progress, the high prevalence of sexual violence among female adolescents reported in this study reinforce the need for heightened action to eliminate gender-based violence in El Salvador.

Female students were also more likely than male students to report feelings of sadness and hopelessness, suicidal ideation and suicidal attempt–a finding that follows similar gender patterns exhibited in the U.S. adolescent population [2,6] and that coincides with the significantly higher rates of depression found for female adolescents in Canada, Great Britain and the United States [22]. Gender differences in suicidal ideation and suicide attempt identified in these students also mirror the broader epidemiologic profile of El Salvador [10], Guatemala [23] and Nicaragua [24] in terms of the higher suicide rates reported for adolescent females. Gender differences in emotional and mental health found in this study may speak to important gender inequities in El Salvador, underscoring the need for an increased gender focus in health research and intervention.

We found a similar lifetime tobacco prevalence use and marijuana use as reported for Salvadoran adolescents (mean age = 16 years, n = 1,628) in the PACARDO study [14]. The relatively high percentage of students reporting cigarette use along with the lack of statistically significant difference for binge drinking between male and female students suggests that these substances may be the more socially accepted among both genders than marijuana use. The higher prevalence of tobacco and alcohol use as compared to other drugs mirrors similar findings from a study conducted with youth in the early 1990s in San Salvador [10] as well as recent findings from the PACARDO study [14]. We found that students 15–20 years old were significantly more likely to report current cigarette use and binge drinking than younger students, suggesting the need to enhance prevention efforts prior to and during this developmental age.

Several interesting gender differences in sexual behaviors are worth noting, with perhaps the most striking being the large difference in reporting of sexual experience. Just under half of the male study participants reported having had sexual intercourse as compared with under 8% of females, and males were significantly more likely to report having had sex at or before age of 13 years. One explanation for these differences in sexual behavior is a reporting bias in which males may exaggerate and females under-represent their sexual experiences. Gender differences in the prevalence of sexual intercourse may also be due to a possible cultural acceptance for young males to engage in sexual intercourse at an early age, which is supported by similar findings of sexual experience in male youth in Nicaragua [25,26]. For single females of reproductive age living in El Salvador, on the other hand, premarital sex is not widely accepted. Informal discussions with female and male teachers in El Salvador about the early age of male sexual intercourse revealed that some Salvadorans believe young males need to have sexual intercourse in order to ensure that they mature adequately as males. Formal qualitative research is needed to fully understand premarital sexual behavior in El Salvador.

Lastly, with fairly equal proportions of sexually experienced male and female students reporting no condom use, this finding is of particular concern when considering that El Salvador has one of the highest teenage pregnancy rates in Latin America and the Caribbean [27], ranking fourth out of the eight countries in the Central American region (including Mexico) [28]. With the population aged 15–24 years accounting for 26.7% of the HIV/AIDS cases in El Salvador in 2000 [29], the need for education that promotes safe sexual practices among adolescents is further emphasized. Recognizing that sexual abstinence and the possible use of contraceptive methods may account for the lack of pregnancy experience reported by females in this study, other explanations may include a social reporting bias in which female students are reluctant to share their pregnancy history or a selection bias inherent in the sample resulting from pregnant females dropping out of school or attending schools specifically designated for pregnant teenagers.

### Subjective economic status patterns

Recognizing the limitations of a one-item measure to assess economic status, we did find an inverse association between the subjective economic status measure and lack of safety to go to school and feelings of sadness/hopelessness and suicidal ideation. Although studies in the United States have also found an association between safety perceptions and socioeconomic status [30,31], the associations between depressive symptoms and suicidal ideation with socioeconomic status are less straightforward [32]. In providing insight into the potential role of economic stressors and suicidal ideation, a recent study of U.S. families by Yoder and Hoyt [33] found family economic pressure to be indirectly related to adolescent suicidal ideation through a pathway of parental depressive symptoms, parental hostile behavior and physical abuse, adolescent self-esteem and adolescent depressive symptoms. Further research is needed to understand the association between economic disadvantage and youth health risk factors in El Salvador.

### Urban-rural

Among the more striking findings of this study are the differences in risk behavior between rural and urban secondary students. Overall, urban students were significantly more likely than rural students to report the majority of risk behaviors related to aggression, depressive symptoms and suicidal ideation, and substance use. With the exception of sexual intercourse at or before age 13–which urban males were more likely than rural males to report–sexual behavior was the only risk behavior domain that revealed no statistically significant differences between urban and rural students. These differences are important not only to identify groups for intervention but also to establish a base from which to explore the factors in the rural setting that may protect adolescents against specific health risk behaviors.

### Limitations

This sample was drawn from students studying in the central region of the country, which may not be representative of the country as a whole. In addition, only 50% of youth in El Salvador are reported to be enrolled in secondary schools [34]. Based on these considerations, we need to be cautious about generalizing these prevalence estimates across all Salvadoran adolescents. As with all youth risk behavior studies, social desirability bias is an important consideration in interpreting the results because students may under- or overestimate their involvement in specific risk behaviors. Adolescent health behaviors have also been associated with an adolescent's religiosity [35] and academic performance [36,37], two potential effect modifying/confounding variables that were not examined in this study. Lastly, in light of the increased obesity prevalence rates in Latin America [38], future risk behavior studies should include items on physical activity and nutrition behaviors of Salvadoran adolescents.

## Conclusion

Antonovsky [39] states: "... [W]e are always in the dangerous river of life. The twin question is: *How dangerous is our river? How well can we swim?*" Youth risk behavior studies provide one mechanism for assessing the 'level of danger' in an adolescent's river of life. The high percentage of students engaging in health risk behaviors found in this study, with 20%–30% of students reporting at least one health risk behavior from each of the five domains examined, suggests that enhanced adolescent health promotion efforts are warranted to promote a safer river of life for adolescents living in El Salvador. In order to develop strategies for youth health risk behavior reduction and in meeting calls for stronger adolescent health policies and indicators as put forth by organizations such as the United Nations, a surveillance strategy to measure youth risk behavior change over time is needed.

Future research efforts should explore the underlying factors associated with geographic differences in risk behavior, the important gender differences in risk behaviors– including the factors associated with higher prevalence of depressive symptoms, suicidal ideation, and forced sexual intercourse in females and higher sexual intercourse and substance use in males, and the factors that contribute to the young age of sexual intercourse as well as low condom use. With a growing body of literature that points to the importance of supportive family and school relationships for reducing several youth health risk behaviors [see [40,41], future efforts should also focus on those factors that may exert a protective effect against engagement in youth risk behavior, not only those factors associated with increased risk. Ultimately, exploring the association between protective social resources and health risk behaviors may reveal that the dangers in the adolescent river of life are prevented by the access an adolescent has to the life rafts and buoys that reduce health risk behavior and help him/her to swim.

## Competing interests

The author(s) declare that they have no competing interests.

## Authors' contributions

AS performed the statistical analysis of the data and took the primary role in drafting the manuscript. BS provided important guidance with the data collection strategy and analysis of the data and contributed to the discussion section. SK contributed to the analysis of the data, assisted with the interpretation of the results, and provided critical review of the manuscript. AS and BS conceived of the study, and AS was the principal investigator for the original study on which this paper is based. All authors read and approved the final manuscript.

## Pre-publication history

The pre-publication history for this paper can be accessed here:


